# Translation and Psychometric Properties of the Arabic Version of Michigan Neuropathy Screening Instrument in Type 2 Diabetes

**DOI:** 10.1155/2019/2673105

**Published:** 2019-03-31

**Authors:** Maha T. Mohammad, Jennifer Muhaidat, Munther S. Momani, Lara Al-Khlaifat, Rasha Okasheh, Dania Qutishat, Emad Al-Yahya

**Affiliations:** ^1^Department of Physiotherapy, School of Rehabilitation Sciences, The University of Jordan, Amman, Jordan; ^2^Internal Medicine Department, Jordan University Hospital, The University of Jordan, Amman, Jordan

## Abstract

**Objective:**

To translate the patient questionnaire section of the Michigan Neuropathy Screening Instrument (MNSI) into Arabic, examine the reliability of the translated version, and provide descriptive data on a sample of patients with type 2 diabetes.

**Methods:**

Researchers used the translation-back translation method to obtain MNSI Arabic. The test was then applied on 76 patients with type 2 diabetes. A subgroup of 25 patients answered MNSI Arabic twice to examine reliability.

**Results:**

The intraclass correlation coefficient was 0.87, revealing good reliability of MNSI Arabic. The most common symptoms patients complained of were numbness (62%), prickling feelings (57%), burning pain (47%), and pain with walking (46%).

**Conclusion:**

Similar to the original MNSI version, our study demonstrates that the Arabic version of the MNSI questionnaire is a reliable tool for screening the symptomatic neuropathy status in patients with type 2 diabetes. Availability of this tool in Arabic will provide valuable and easy-to-obtain screening information regarding diabetic peripheral neuropathy that may help delay its complications by promoting early management.

## 1. Introduction

In the recent years, there has been a steady global increase in the prevalence of diabetes mellitus (DM) and, accordingly, its complications [1–4]. The International Diabetes Federation estimated the global diabetes prevalence among adults in the age range 18-99 in 2017 to be 451 million, with a projected increase to 693 million in 2045 [4, 5]. Developing countries in the Middle East and North Africa have had the second-largest increase worldwide in adult diabetes prevalence [2]. Diabetic peripheral neuropathy (DPN) is one of the common and serious complications of DM [6, 7]. Prevalence estimates of DPN vary widely depending on several factors such as the type and duration of DM with prevalence rates approaching 50% after 10 years of diagnosis with DM [6]. Studies from the Middle East region estimated the prevalence of DPN among patients with type 2 DM between 25.6 and 39.5% [8–10]. Neuropathy causes a progressive disturbance in the function of peripheral nerves leading to increased risk of ulcers, infections, and amputations [6, 11, 12], which have a significant effect on patients' quality of life [11]. A major concern with DPN is that it may be asymptomatic, hence undiagnosed, in a considerable number of patients [6, 13, 14]. Early screening of DPN may help delay its complications by initiating preventive therapies such as intensive glycemic control, foot management, or physical activity and exercise [6, 12, 15–18].

Several assessment tools are available for the screening and diagnosis of DPN. Nerve conduction studies (NCS) are usually considered the gold standard to establish a confirmed diagnosis of DPN [7, 12, 19]. Albeit objective and sensitive, this tool is expensive, requires specialized personnel, and is not readily available in most clinical practices [19]. Therefore, NCS are rarely done except in atypical cases [6, 13].

Alternative, and more clinically available, assessment methods for DPN include Semmes Weinstein monofilament examination (SWME) [19–21], vibration perception threshold [20, 22], physical exam [13], and questionnaires such as the Michigan Neuropathy Screening Instrument (MNSI) [23]. Current American Diabetes Association guidelines recommend that patients undergo annual screening to detect advanced neuropathy and feet at risk of ulceration using 10-gram SWME [13]. However, the onset of ulcers could occur so rapidly that an annual exam may not suffice, hence the need for more practical handy tool to assess DPN that physicians could apply quickly during regular patient checkup visits.

Feldman et al. developed MNSI to provide a simple validated screening tool for DPN [23, 24]. The test comprises 2 parts; a 15-item questionnaire and a physical exam. Sensitivity and specificity of the test are 80 and 95%, respectively [23]. The test is simple and can be applied in routine clinical setting by the wide range of health professionals who deal with patients with DM. Since its publication, MNSI has been used in a wide range of literature on DM to confirm the diagnosis of DPN [25–28].

In Arabic-speaking countries, there is a need for a similar assessment tool that can be used easily and quickly in the clinical setting to provide physicians with information about the risk of DPN in their patients. The aim of the current study was to translate and examine the reliability of the Arabic version of the questionnaire section of the MNSI. In addition, descriptive data on MNSI for a sample of patients seen in a diabetes clinic are provided.

## 2. Methods

### 2.1. Translation of MNSI

Translation process followed the translation-back translation method. First, authors obtained permission from Dr. Feldman (tool developer) to perform the translation. Following Dr. Feldman's approval, two authors (MM and EA) who are fluent in both Arabic and English separately performed the forward translation. This produced 2 Arabic versions of the tool that were combined in a pooled version after a meeting between the authors to discuss differences between the 2 versions. Two native English speakers—not healthcare individuals—but fluent in Arabic and with no prior knowledge of the MNSI performed the backward translation which yielded a version equivalent to the original English one. Accordingly, no changes to the Arabic version were made. Following translation of the tool, the researchers interviewed a sample of 5 patients with DM in one-on-one interviews to discuss the Arabic version and check the clarity of items. Patients suggested alternative words for 2 items which were added to the questionnaire producing the final version of MNSI Arabic (Supplementary Materials (available here)).

### 2.2. Participants

Researchers recruited a convenience sample of patients with type 2 DM only. Recruitment took place while patients were waiting for their physician appointment at the diabetes clinic. In order to improve the generalizability of the study results, researchers approached all patients visiting the clinic and kept inclusion/exclusion criteria to a minimum. Inclusion criteria were age > 18 years and a confirmed diagnosis of type 2 DM. Physicians established the diagnosis according to the World Health Organization diagnostic criteria (fasting plasma glucose ≥ 7.0 mmol/l (126 mg/dl)) [29]. Exclusion criteria were other differential diagnoses of peripheral neuropathy including stroke, peripheral vascular disease, vitamin B12 deficiency, and hypothyroidism. The researchers obtained information on those disorders through patients' files and history. The Institutional Review Board at Jordan University Hospital approved the study procedures, and all participants provided written informed consent before taking part in the study. All study procedures were in accordance with the Declaration of Helsinki principles.

### 2.3. Procedure

Firstly, patients filled in the MNSI Arabic. The questionnaire was self-administered and took approximately 5 minutes. It consists of 15 yes-no questions on symptoms such as numbness, burning pain, open sores, night symptoms, temperature sensation, or pain with walking. Then, a researcher applied the physical exam component of MNSI. The physical exam includes observation of feet appearance, presence of ulcers, ankle reflexes, vibration sensation, and monofilament exam. For the reliability part, researchers called a random subsample of patients few days later and asked them the MNSI questions over the phone.

### 2.4. Statistical Analysis

Descriptive statistics were calculated using means and standard deviations for continuous measures and frequencies and percentages for count data. Test-retest reliability was calculated using intraclass correlation coefficient (ICC model 3,1). Cronbach's alpha was used to evaluate the internal consistency of the questionnaire, the physical exam, and the two parts combined.

The required sample size was determined based on Hobart et al. who reported that a minimum sample size of 20 provides representative estimates for reliability and internal consistency measures [30].

Statistical analysis was done using SPSS, version 22 (SPSS Inc., Chicago, Illinois). Statistical level of significance was set at *α* < 0.05.

## 3. Results

### 3.1. Patient Characteristics

A total of 76 patients with type 2 diabetes participated in the study. The average (SD) age for the sample was 59.8 (10) years. The majority of the patients (*n* = 62) were females. The mean (SD) duration of type 2 diabetes was 9.3 (7.3) years. Twenty-five patients completed MNSI Arabic questionnaire twice for the reliability part. The retest was conducted between 2 and 10 days after the initial assessment. The average (SD) age of patients was 59.1 (11) years; the majority (*n* = 22) were females, and they had DM for an average (SD) of 9.7 (9.3) years. Characteristics of patients who participated in the study are summarized in Table 1.

### 3.2. Psychometric Data

When examining the test-retest reliability of the MNSI Arabic questionnaire, ICC value was 0.87 (95% confidence interval = 0.70–0.94) (*p* < 0.001). The value of Cronbach's alpha for the questionnaire, physical exam, and both parts combined was 0.74, 0.73, and 0.78, respectively, indicating high internal consistency of all components of the tool. Individual items showed good internal consistency in the questionnaire and the physical exam; Cronbach's alphas were >0.699 and 0.697, respectively.

### 3.3. Descriptive Data

On MNSI, the average (SD) score on the questionnaire was 3.5 (2.3) and on physical exam was 2.4 (1.8). Using cutoff scores ≥ 4 for the questionnaire and ≥2.5 for the physical exam parts of MNSI [31] showed that 37 patients (48.7%) had abnormal scores on MNSI questionnaire and 34 patients (44.7%) had abnormal scores on MNSI physical exam. The most common symptoms patients complained of were numbness (62%), prickling feelings (57%), burning pain (47%), and pain while walking (46%). On physical exam, the most common abnormalities observed were in ankle reflexes and vibration perception which were absent bilaterally in 42% and 28% of the patients, respectively.

## 4. Discussion and Conclusion

Early diagnosis of DPN is of paramount importance to initiate management and possibly prevent or delay future complications. In Arab countries, despite the increasing prevalence of DM [1, 32], scarce evidence is available regarding the prevalence of DPN and its consequences [8–10, 33]. This in part may be due to the difficulty associated with performing the gold standard test (nerve conduction studies) in the clinical settings and the unavailability of simpler tools (such as MNSI) in Arabic language. Historically, clinicians relied on patient symptoms for the diagnosis of DPN, with the resultant oblivion of asymptomatic cases. This study provides a simple and quick tool that physicians and health professionals can use to screen for DPN using patient symptoms and physical exam.

The current study translated the patient questionnaire section of MNSI to Arabic using the forward-backward translation method. Only the questionnaire section was translated because in most Arab countries health professionals are educated and trained in the English language, so they could read and apply the physical exam component of MNSI. The need for MNSI Arabic arises when performing the questionnaire section of the tool with patients. Results suggest that the MNSI Arabic questionnaire is a reliable screening tool for DPN in symptomatic patients with type 2 DM. The high ICC value (0.87) indicates that the tool provides consistent measurements overtime. High Cronbach's alpha (>0.73) reflects the homogenous structure of items in the tool and the homogeneity between the questionnaire and the physical exam parts.

Filling in the questionnaire took around 5 minutes of patients' time; it could be done while the patient is waiting for their healthcare professional appointment. With increasing work load on physicians—whereby visit length varies among countries with an estimate of 10 minutes per visit [34]—and decreasing amount of time spent with patients, a tool such as MNSI could be easily applied in the busy clinical setting.

Screening for DPN should be a part of the regular clinical exam for patients with DM. While patients may not volunteer symptoms immediately during the physician visit [6], the use of the MNSI Arabic questionnaire will help raise awareness among patients by teaching them which symptoms to consider on their own. Notwithstanding, attention should be paid to the fact that neuropathy may be asymptomatic in up to 50% of patients. Physicians should always complement the questionnaire with the physical exam part of MNSI.

Our results show that numbness, prickling feeling, burning pain, and pain while walking are the most common symptoms patients have. Several patients, however, commented that their pain while walking was related to knee arthritis; so this particular symptom needs to be considered along with the other symptoms for the diagnosis of DPN. Baraz et al. reported a similar pattern of symptoms in patients with DM; the most common symptoms they reported were paresthesia (72%), pain at feet (71%), pins and needles at feet tips (71%), numbness (63%), and coldness at feet (51%) [21].

One of the limitations of the current study is the need to validate Arabic MNSI against NCS, which is considered the gold standard for diagnosis of DPN [7, 19]. However, NCS is not a routine part of clinical testing in a diabetes clinic such as the one where this study was conducted. In addition, the original research study that developed the MNSI validated it against NCS and reported excellent sensitivity and specificity. Another limitation is that the majority of patients who participated in the reliability assessment were females. However, there is no reason to suspect that the reliability of the responses to the questionnaire is gender-biased, although females tend to report more frequent and intense symptoms than males in general [35].

With the recent epidemic of DM in developing countries, there is a high need for screening tools for its complications including DPN. The availability of MNSI Arabic should facilitate early management of DPN and future research on this important complication of the disease. Understanding the health burden, disability, and implications on the quality of life resulting from DPN are examples of benefits of such studies. In addition, future studies should examine the psychometric properties of MNSI with patients with type 1 DM.

In conclusion, MNSI Arabic is a reliable tool that can be used in the clinical setting by a wide range of health professionals working with patients with type 2 diabetes to provide quick screening for the presence of peripheral neuropathies in symptomatic patients. With proper education, patients can use their symptoms to monitor the progression of DPN. Numbness, prickling feelings, and burning pain are the most common symptoms patients have.

## Figures and Tables

**Table 1:** Patient demographics and neuropathy assessment results.

	Patients (*n* = 76)	Patients who completed the reliability part (*n* = 25)	
Age (mean ± SD, range)	59.8 ± 10, 28–81	59.1 ± 11, 38–81	
Gender: female (*n* (%))	62 (82%)	22 (88%)	
Duration since diabetes: years (mean ± SD)	9.3 ± 7.3	9.7 ± 9.3	
HbA1c (mmol/l) (mean ± SD)	7.5 ± 1.5	7.8 ± 1.8	
MNSI Arabic questionnaire score (mean ± SD)	3.5 ± 2.3	First assessment	Second assessment
		4.4 ± 2.6	4.1 ± 2.5
Abnormal MNSI Arabic questionnaire (score ≥ 4) (*n* (%))	37 (48.7%)	15 (60%)	
MNSI physical exam score (mean ± SD)	2.4 ± 1.8	2.7 ± 1.7	
Abnormal MNSI physical exam score (score ≥ 2.5) (*n* (%))	34 (44.7%)	13 (52%)	

SD: standard deviation; DPN: diabetic peripheral neuropathy; MNSI: Michigan Neuropathy Screening Instrument; HbA1c: hemoglobin A1c.

## Data Availability

The data used to support findings of this study can be obtained by sending requests to the corresponding author. Requests will be considered upon Institutional Review Board approval.

## Supplementary Materials

Supplementary Materials Final Arabic version of the Michigan Neuropathy Screening Instrument (MNSI Arabic).

## References

[1] WHO (2016). *Global Report on Diabetes*.

[2] NCD Risk Factor Collaboration (NCD-RisC) (2016). Worldwide trends in diabetes since 1980: a pooled analysis of 751 population-based studies with 4.4 million participants. *The Lancet*.

[3] Mathers C. D., Loncar D. (2006). Projections of global mortality and burden of disease from 2002 to 2030. *PLoS Medicine*.

[4] International Diabetes Federation IDF diabetes atlas. https://www.diabetesatlas.org/.

[5] Cho N. H., Shaw J. E., Karuranga S. (2018). IDF diabetes atlas: global estimates of diabetes prevalence for 2017 and projections for 2045. *Diabetes Research and Clinical Practice*.

[6] Pop-Busui R., Boulton A. J. M., Feldman E. L. (2016). Diabetic neuropathy: a position statement by the American Diabetes Association. *Diabetes Care*.

[7] Tesfaye S., Boulton A. J. M., Dyck P. J. (2010). Diabetic neuropathies: update on definitions, diagnostic criteria, estimation of severity, and treatments. *Diabetes Care*.

[8] Khawaja N., Abu-Shennar J., Saleh M., Dahbour S. S., Khader Y. S., Ajlouni K. M. (2018). The prevalence and risk factors of peripheral neuropathy among patients with type 2 diabetes mellitus; the case of Jordan. *Diabetology & Metabolic Syndrome*.

[9] Al-Mahroos F., Al-Roomi K. (2007). Diabetic neuropathy, foot ulceration, peripheral vascular disease and potential risk factors among patients with diabetes in Bahrain: a nationwide primary care diabetes clinic-based study. *Annals of Saudi Medicine*.

[10] Al-Kaabi J. M., Al Maskari F., Zoubeidi T. (2014). Prevalence and determinants of peripheral neuropathy in patients with type 2 diabetes attending a tertiary care center in the United Arab Emirates. *Journal of Diabetes & Metabolism*.

[11] Boulton A. J. M., Kirsner R. S., Vileikyte L. (2004). Neuropathic diabetic foot ulcers. *The New England Journal of Medicine*.

[12] Bansal V., Kalita J., Misra U. K. (2006). Diabetic neuropathy. *Postgraduate Medical Journal*.

[13] American Diabetes Association (2016). 10. Microvascular complications and foot care. *Diabetes Care*.

[14] Ziegler D., Landgraf R., Lobmann R. (2018). Painful and painless neuropathies are distinct and largely undiagnosed entities in subjects participating in an educational initiative (PROTECT study). *Diabetes Research and Clinical Practice*.

[15] Dy S. M., Bennett W. L., Sharma R. (2017). Preventing complications and treating symptoms of diabetic peripheral neuropathy. Comparative Effectiveness Review No. 187.

[16] Diabetes Control and Complications Trial Research Group, Nathan D. M., Genuth S. (1993). The effect of intensive treatment of diabetes on the development and progression of long-term complications in insulin-dependent diabetes mellitus. *The New England Journal of Medicine*.

[17] Albers J. W., Herman W. H., Pop-Busui R. (2010). Effect of prior intensive insulin treatment during the Diabetes Control and Complications Trial (DCCT) on peripheral neuropathy in type 1 diabetes during the Epidemiology of Diabetes Interventions and Complications (EDIC) study. *Diabetes Care*.

[18] Matos M., Mendes R., Silva A. B., Sousa N. (2018). Physical activity and exercise on diabetic foot related outcomes: a systematic review. *Diabetes Research and Clinical Practice*.

[19] Feng Y., Schlosser F. J., Sumpio B. E. (2009). The Semmes Weinstein monofilament examination as a screening tool for diabetic peripheral neuropathy. *Journal of Vascular Surgery*.

[20] Perkins B. A., Olaleye D., Zinman B., Bril V. (2001). Simple screening tests for peripheral neuropathy in the diabetes clinic. *Diabetes Care*.

[21] Baraz S., Zarea K., Shahbazian H., Latifi S. (2014). Comparison of the accuracy of monofilament testing at various points of feet in peripheral diabetic neuropathy screening. *Journal of Diabetes and Metabolic Disorders*.

[22] Valk G. D., de Sonnaville J. J. J., van Houtum W. H. (1997). The assessment of diabetic polyneuropathy in daily clinical practice: reproducibility and validity of Semmes Weinstein monofilaments examination and clinical neurological examination. *Muscle & Nerve*.

[23] Feldman E. L., Stevens M. J., Thomas P. K., Brown M. B., Canal N., Greene D. A. (1994). A practical two-step quantitative clinical and electrophysiological assessment for the diagnosis and staging of diabetic neuropathy. *Diabetes Care*.

[24] Feldman E. L., Stevens M. J. (1994). Clinical testing in diabetic peripheral neuropathy. *The Canadian Journal of Neurological Sciences*.

[25] Sacco I. C. N., Picon A. P., Macedo D. O., Butugan M. K., Watari R., Sartor C. D. (2015). Alterations in the lower limb joint moments precede the peripheral neuropathy diagnosis in diabetes patients. *Diabetes Technology & Therapeutics*.

[26] Camargo M. R., Barela J. A., Nozabieli A. J. L., Mantovani A. M., Martinelli A. R., Fregonesi C. E. P. T. (2015). Balance and ankle muscle strength predict spatiotemporal gait parameters in individuals with diabetic peripheral neuropathy. *Diabetes & Metabolic Syndrome: Clinical Research & Reviews*.

[27] Jaiswal M., Divers J., Dabelea D. (2017). Prevalence of and risk factors for diabetic peripheral neuropathy in youth with type 1 and type 2 diabetes: SEARCH for diabetes in youth study. *Diabetes Care*.

[28] Didangelos T., Tziomalos K., Margaritidis C. (2017). Efficacy of administration of an angiotensin converting enzyme inhibitor for two years on autonomic and peripheral neuropathy in patients with diabetes mellitus. *Journal Diabetes Research*.

[29] Alberti K. G. M. M., Zimmet P. Z. (1998). Definition, diagnosis and classification of diabetes mellitus and its complications. Part 1: diagnosis and classification of diabetes mellitus provisional report of a WHO consultation. *Diabetic Medicine*.

[30] Hobart J. C., Cano S. J., Warner T. T., Thompson A. J. (2012). What sample sizes for reliability and validity studies in neurology?. *Journal of Neurology*.

[31] Herman W. H., Pop-Busui R., Braffett B. H. (2012). Use of the Michigan Neuropathy Screening Instrument as a measure of distal symmetrical peripheral neuropathy in type 1 diabetes: results from the Diabetes Control and Complications Trial/Epidemiology of Diabetes Interventions and Complications. *Diabetic Medicine*.

[32] Ajlouni K., Khader Y. S., Batieha A., Ajlouni H., el-Khateeb M. (2008). An increase in prevalence of diabetes mellitus in Jordan over 10 years. *Journal of Diabetes and its Complications*.

[33] Kandil M. R., Darwish E. S., Khedr E. M., Sabry M. M., Abdulah M. A. (2013). A community-based epidemiological study of peripheral neuropathies in Assiut, Egypt. *Neurological Research*.

[34] Dugdale D. C., Epstein R., Pantilat S. Z. (1999). Time and the patient-physician relationship. *Journal of General Internal Medicine*.

[35] Van den Bergh O., Witthöft M., Petersen S., Brown R. J. (2017). Symptoms and the body: taking the inferential leap. *Neuroscience & Biobehavioral Reviews*.

