# Supplementary material for: Translation and Psychometric Properties of the Arabic Version of Michigan Neuropathy Screening Instrument in Type 2 Diabetes
Source: J Diabetes Res. 2019 Mar 31;2019:2673105. doi: 10.1155/2019/2673105 (PMC6462346; doi:10.1155/2019/2673105)
Supplement: Supplementary Materials — Final Arabic version of the Michigan Neuropathy Screening Instrument (MNSI Arabic). [file 2673105.f1.pdf]

Supplementary Material

التاريخ ___________

نسخة المريض

أداة متشغان لفحص الاعتلال العصبي

التاريخ المرضي (يتم تعبئته من قبل الشخص المصاب بمرض السكري):

يرجى التكرم بالإجابة عن الأسئلة التالية حول الإحساس في ساقيك وقدميك. أجب بنعم أو لا بناءً على ما تشعر به عادة وليس بالضرورة ما تشعر به الآن. شكراً.

1. هل تشعر بخدر في ساقيك و/أو قدميك؟	☐ نعم	☐ لا
2. هل تشعر بألم حارق (نار) في ساقيك و/أو قدميك؟	☐ نعم	☐ لا
3. هل قدماك حساستان بشكل زائد للمس؟	☐ نعم	☐ لا
4. هل تشعر بتشنجات عضلية (شد عضلي) في ساقيك و/أو قدميك؟	☐ نعم	☐ لا
5. هل تشعر بإحساس النمنمة (التنميل) في ساقيك و/أو قدميك؟	☐ نعم	☐ لا
6. هل تشعر بالألم عندما تلمس أغطية السرير جلدك؟	☐ نعم	☐ لا
7. عند الاستحمام, هل تستطيع التمييز بين الماء الحار والماء البارد؟	☐ نعم	☐ لا
8. هل سبق وأن أصبت بتقرح في قدمك؟	☐ نعم	☐ لا
9. هل سبق وأن أخبرك طبيبك بأن لديك الاعتلال العصبي السكري (القدم السكري)؟	☐ نعم	☐ لا
10. هل تشعر بضعف عام أغلب الوقت؟	☐ نعم	☐ لا
11. هل تزداد أعراضك سوءا في الليل؟	☐ نعم	☐ لا
12. هل تؤلمك ساقاك عندما تمشي؟	☐ نعم	☐ لا
13. هل أنت قادر على الإحساس بقدميك عندما تمشي؟	☐ نعم	☐ لا
14. هل جلد قدميك جاف لدرجة أنه يصاب بتشققات؟	☐ نعم	☐ لا
15. هل سبق وأن أجريت عملية بتر؟	☐ نعم	☐ لا

المجموع: __________
